# Characteristics of spinal microglia in aged and obese mice: potential contributions to impaired sensory behavior

**DOI:** 10.1186/s12979-015-0049-5

**Published:** 2015-11-24

**Authors:** SeungHwan Lee, YaSi Wu, Xiang Qun Shi, Ji Zhang

**Affiliations:** The Alan Edwards Centre for Research on Pain, McGill University, 740 Docteur Penfield Ave, Suite 3200C, Montreal, QC H3A 0G1 Canada; Department of Neurology & Neurosurgery, Faculty of Medicine, McGill University, Montreal, QC H3A 2B4 Canada; Faculty of Dentistry, McGill University, Montreal, QC H3A 0C7 Canada

**Keywords:** Aging, Obesity, Microglia, Pain, Spinal cord, Inflammation

## Abstract

**Background:**

Both aging and obesity have been recognized widely as health conditions that profoundly affect individuals, families and the society. Aged and obese people often report altered pain responses while underlying mechanisms have not been fully elucidated. We aim to understand whether spinal microglia could potentially contribute to altered sensory behavior in aged and obese population.

**Results:**

In this study, we monitored pain behavior in adult (6 months) and aged (17 months) mice fed with diet containing 10 % or 60 % Kcal fat. The group of young adult (3 months) mice was included as theoretical baseline control. Compared with lean adult animals, diet-induced-obese (DIO) adult, lean and DIO-aged mice showed enhanced painful response to heat and cold stimuli, while exhibiting hyposensitivity to mechanical stimulation. The impact of aging and obesity on microglia properties was evidenced by an increased microglial cell density in the spinal cords, stereotypic morphological changes and polarization towards pro-inflammatory phenotype. Obesity strikingly exacerbated the effect of aging on spinal microglia.

**Conclusion:**

Aging/obesity altered microglia properties in the spinal cords, which can dysregulate neuron-microglia crosstalk and impair physiological pain signal transmission. The inflammatory functions of microglia have special relevance for understanding of abnormal pain behavior in aged/obese populations.

## Background

People are living longer. The number of older persons has tripled over the last 50 years; it will be more than triple again over the next 50 years [1]. We expect to have more than 20 % of the population over 65 years old by 2050 [2]. In parallel, obesity has become a global epidemic. From 2007 to 2009, the prevalence of obesity was 24.1 % in Canada and 34.4 % in the United States [3]. The number of obese old adults is strongly increasing over time [4]. As a result of the gradual, lifelong accumulation of a wide variety of molecular and cellular damages, old people usually cope with a progressive decline in their physical and cognitive functions. Pain is shown to be a common problem where prevalence rates can reach over 61 % among older adults [5, 6]. Obese elders are at increased odds to develop pain [7].

The mechanisms underlying chronic pain in the elderly are not fully understood, especially in obese elders. Because both aging and obesity are associated with a low grade chronic pro-inflammation [8–10], and inflammation contributes to the development of abnormal painful behavior [11, 12], inflammation could be one of the causal pathways for altered pain behavior in aging and obesity. As resident immune cells of the central nervous system (CNS), microglia perform functions similar to those of tissue macrophages in other organs; they constitute the first line of defense against any disturbance of local homeostasis [13]. Microglia have been recognized as orchestrators of the CNS inflammatory response in various disease conditions. However, microglia biology and function are largely altered in aged brain [14]. Their involvement in the maintenance of local homeostasis can vary within a continuum of states from beneficial to detrimental for neuronal function and survival [15]. Unlike the effects of aging, the impact of obesity on microglia have been much less well investigated, however, some recent studies highlighted the potential influence of metabolic disorders, including obesity on microglia properties [16, 17].

The spinal cord is the first relay site in the transmission of nociceptive information from the periphery to the brain. Spinal microglia become activated rapidly following an injury to peripheral nerve. The contribution of activated spinal microglia in the generation and maintenance of injury triggered chronic pain has been well established [18, 19]. It is thus of importance to understand how aging and obesity affect spinal microglia phenotypes and function, and whether such changes could be related to abnormal sensory behavior in elderly. It will be intriguing to further understand whether and how obesity can impact spinal microglia, with or without priming by aging. In this study, by using C57BL/6 mice fed with regular or high fat diet, we characterized the phenotypes of spinal microglia in adult (6 months old) lean and obese mice, as well as in aged (17 months old) lean and obese mice. A special attention was paid to microglia inflammatory properties to understand their potential contribution to aging and/or obesity associated impaired sensory behavior in these animals.

## Results

### Impaired motor and sensory functions in adult obese, aged and aged-obese mice

**Fig. 1:**
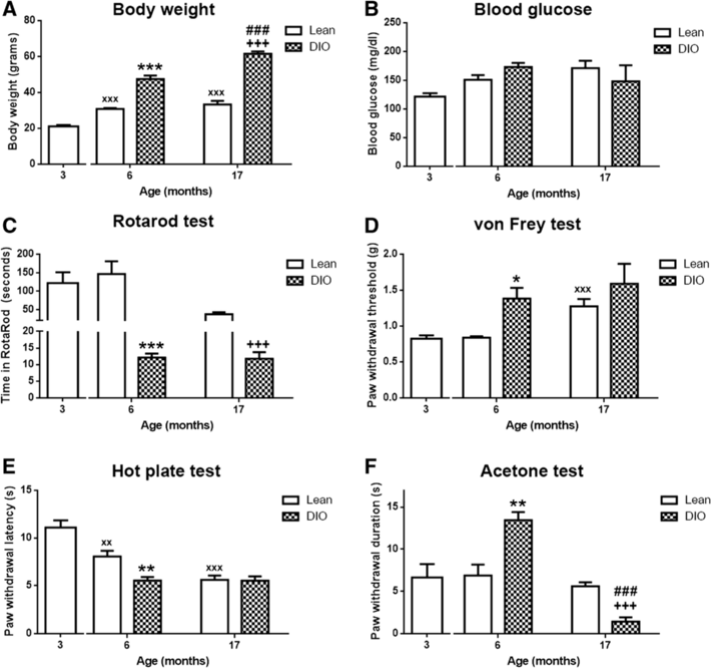
Impaired motor and sensory functions in adult obese, aged and aged obese mice. **a**) Body weight changes were observed in DIO mice, significantly increased in both DIO 6 and 17 month-mice compared to age-matched lean mice. (*n* = 6–8/group, ***p < 0.001, lean 6 vs. DIO 6, +++*p* < 0.001, lean 17 vs. DIO 17, ###*p* < 0.001, DIO 6 vs. DIO 17, ^xxx^ *p* < 0.001, lean 3 vs. lean 6, 17). **b**) Blood glucose levels were not significantly altered after long term high-fat diet (*n* = 6–8/group). **c**) Impaired motor coordination was observed in both DIO 6 and 17 month-mice, while lean 17 month-mice stayed less time on Rotarod. (*n* = 6–8/group, ***p < 0.001, lean 6 vs. DIO 6, +++*p* < 0.001, lean 17 vs. DIO 17,) **d**) Mechanical allodynia was reversed with aging (^xxx^ *p* < 0.001, lean 3 vs. lean17) and with obesity in 6 month-mice (*n* = 6–8/group, *p < 0.05, lean 6 vs. DIO 6). **e**) Thermal hypersensitivity was observed in DIO 6 month-mice compared to lean 6 month-mice (*n* = 6–8/group, **p < 0.01, lean 6 vs. DIO 6) as well as in aged mice (*n* = 6–8/group, ^xx^ *p* < 0.01, ^xxx^ *p* < 0.001, lean 3 months vs. lean 6, 17 months). **f**) Cold hypersensitivity was detected in DIO 6 month-mice (*n* = 6–8/group, **p < 0.01, lean 6 vs. DIO 6), however, it was significantly reversed in DIO 17 month-mice (*n* = 6–8/group, +++*p* < 0.001, lean 17 vs. DIO 17, ###*p* < 0.001, DIO 6 vs. DIO 17)

Diet-induced obese (DIO) mice and lean mice were generated by feeding male C57/BL6 mice with diet containing either 60 % or 10 % fat, starting from 3 months old until the end of the experiments (6 or 17 months). As depicted in Fig. 1a, while the body weights of lean 3 month-mice were 21.13 ± 0.72 g, those of lean 6 month and 17 month-mice were 31.02 ± 0.48 g and 33.51 ± 2.08 g (*p* < 0.001), respectively. DIO-mice weighed 47.71 ± 2.02 g at the age of 6 months and 61.67 ± 1.34 g at 17 months, which was significantly higher than age matched lean controls (*p* < 0.001, vs. lean 6 month and 17 month-mice, respectively). In general, the impact of aging on body weight was significantly higher in obese mice than in lean mice. Long term (either 3 months or 14 months) high fat diet did not induce overt diabetes as no significant difference in blood glucose levels was detected among groups (Fig. 1b). Nevertheless, obesity and aging largely impaired mouse motor coordination. To compare with observations regarding lean 3 month-mice (122.46 ± 28.84 s), lean 6 month-mice spent similar time on Rotarod (149 ± 32.90 s) whereas lean 17 month-mice spent much less time on Rotarod (47.5 ± 5.19 s). Almost all obese mice failed in Rotarod test. DIO 6 month-mice stayed on Rotarod for only12.25 ± 1.16 s; and DIO 17 month-mice for 11.83 ± 2.01 s (*p* < 0.001, vs. age-matched lean mice) (Fig. 1c).

To evaluate the effects of obesity and aging on sensory behavior, von Frey hairs, acetone and hot plate tests were used to test mouse responses to mechanical, cold and heat stimulations respectively. Withdrawal thresholds to von Frey hairs were higher in lean 17 month-mice (*p* < 0.001, 1.28 ± 0.10 g) compared to those of lean 3 month-mice, and those of DIO 6 month-mice (*p* < 0.05, 1.38 ± 0.15 g) were also increased compared to age-matched control. In addition, DIO 17 month-mice (1.60 ± 0.28 g) exhibited higher withdrawal thresholds (Fig. 1d). This result indicates that obese and aged mice developed mechanical hyposensitivity. Interestingly, both obese and aged mice demonstrated hypersensitivity to heat stimulation. In the hot plate test, the withdrawal latencies from lean 6 month- and 17 month-mice were 8.08 ± 0.06 s (*p* < 0.01) and 5.65 ± 0.45 s (*p* < 0.001), respectively, while lean 3 month-mice could sustain 55 °C heat stimulation for 11.08 ± 0.74 s. In DIO mice, the withdrawal latency was significantly lower, 5.57 ± 0.39 s (6 months) and 5.56 ± 0.44 s (17 months) (*p* < 0.01, vs. lean 6 month-mice) (Fig. 1e). Whilst aging did not alter mouse response to cold stimulation (lean 3 month-mice: 6.64 ± 1.59 s, lean 6-month mice: 6.90 ± 1.31 s, lean 17 month-mice 5.65 ± 0.45 s), cold hypersensitivity was detected in DIO 6 month-mice, as their withdrawal duration in acetone test was 13.48 ± 0.96 s (*p* < 0.01, vs. lean 6 month-mice). However, the withdrawal duration in acetone test for DIO 17 month-mice was surprisingly low (1.43 ± 0.51 s) (Fig. 1f), although we cannot exclude biased read-out due to technical flaws of the acetone test in these extremely fat mice. It is possible that due to excess weight, their muscle strength was not strong enough to hold up the paw for extended periods of time and their paw withdrawal reactions could be restricted by the size of cubicle containers.

### Impact of aging and obesity on microglia properties and phenotypes

**Fig. 2:**
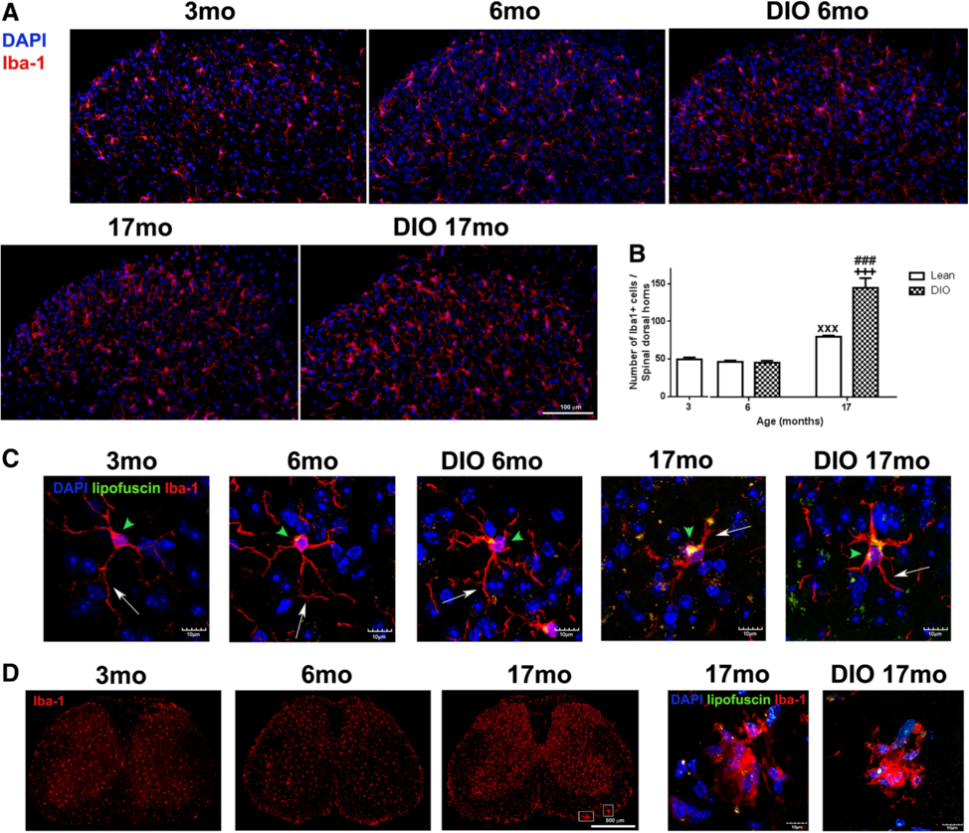
Alterations on spinal microglia with aging and obesity. **a**) Microglia in the dorsal horn of lumbar spinal cord was labeled with Iba1 (red). While there was no obvious difference among the groups of 3 mo, 6 mo and DIO 6 mo mice, the density of Iba1^+^ microglia was higher in lean and DIO 17 month-mice. **b**) Total number of Iba1+ cells was significantly increased in lean 17 month-mice compared to lean 3 month-mice (^xxx^ *p* < 0.001, lean 3 vs. lean 17) which was further increased in DIO 17 month-mice (*n* = 6–10 section/animal, *n* = 3 animals/group, +++*p* < 0.001, lean 17 vs. DIO 17, ###*p* < 0.001, DIO 6 vs. DIO 17). **c**) Iba1^+^ microglia (red) underwent morphological changes with aging and obesity. It was observed that cell bodies were enlarged (arrowheads) and processes were retracted (arrows) while more lipofuscin granules (yellow) were accumulated in these enlarged cell bodies with aging and obesity. **d**) Microglia clusters were found in the spinal cord white matter of aged mice (square in lean 17 month-mice), not found in lean 3 and 6 month-mice. Confocal scanning images showed that clusters were composed of 5-7 microglia and contained less lipofuscin granules

Spinal microglia were immunolabeled by an antibody against Iba-1, a calcium-binding protein specifically expressed in microglia. Representative examples of lumbar spinal cord dorsal horns from all five groups of mice revealed an increase of Iba-1^+^ cell density in lean 17 month- and DIO 17 month- mice (Fig. 2a). Quantitative analysis (Fig. 2b) demonstrated that the number of spinal microglia were similar in lean 3 month- (49.50 ± 2.50), lean 6 month- (46.10 ± 1.71) and DIO 6 month- (45.07 ± 2.09) mice. However, aging significantly increased the number of Iba-1^+^ cells in the spinal cords. Compared to lean 3 month-mice, the number of microglia in lean 17 month-mice reached 79.00 ± 1.46 (*p* < 0.001). While obesity did not really affect the total number of spinal microglia in adult mice, it exacerbated strikingly the effect of aging on microglia. Microglial cell density was increased to two times higher in DIO 17 month-mice (143.00 ± 12.94, *p* < 0.001), comparing with that in DIO 6 month-mice (45.07 ± 2.09) and in lean 3 months (49.50 ± 2.50). The impact of aging and obesity on microglia was also characterized by their morphological changes (Fig. 2c). Microglia in lumbar spinal cords of lean 3 and 6 month-mice displayed small cell bodies with long, thin branching processes, while microglia in DIO 6 month-mice exhibited thicker and rigid branches. In lean and obese 17 month aged mice, microglia had enlarged cell bodies, retracted and thick processes. Their cell bodies were usually filled with a large amount of autofluorescent lipofuscin pigment granules. These undegradable residues of lysosomal digestion have been recognized as hallmark of aging [20]. In addition to that, microglia clusters composed of 4-8 Iba-1^+^ cells were observed in both lean and DIO17 month-mice. They were in amoeboid shape or contained very few, short and highly thickened processes. They were located mainly in white matter of spinal cords, with much less lipofuscin accumulation (Fig. 2d).

**Fig. 3:**
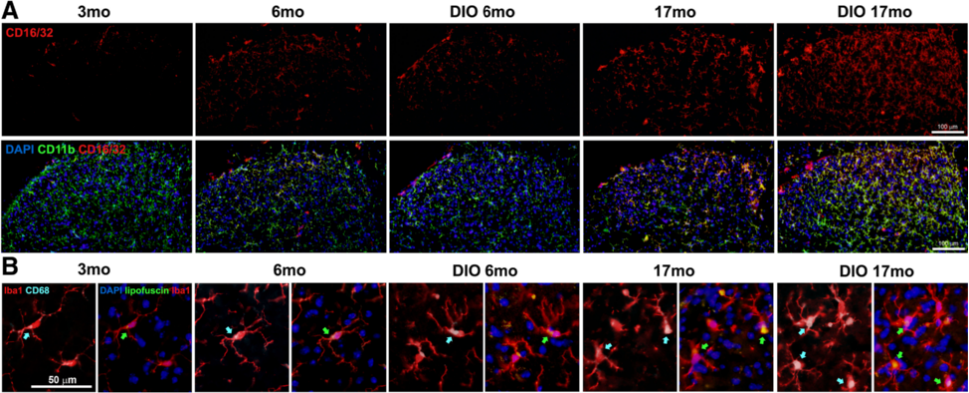
Functional analysis of spinal microglia with aging and obesity. **a**) Expression of FcγR III/II (CD16/32) on CD11b^+^ microglia in the spinal cords. CD16/32 (red) expression was found in dorsal horns of lumbar spinal cords in aged animals (lean and DIO 17 month-mice), which was colocalized with CD11b^+^ microglia (green). Only few CD16/32^+^/CD11b + microglia was observed in lean 3, 6, and DIO 6 month-mice. **b**) CD68 expression was closely associated with lipofuscin accumulation in aged microglia. Arrows indicate CD68 staining (blue, left) and lipofuscin pigments (green, right). The increased lipofuscin deposit with aging (lean 17 and DIO 17 month-mice) was closely associated with CD68. CD68 signals in obese and aged mice anatomically matched with intracellular aggregation of lipofuscin

To define functional phenotypes of microglia in aged and obese mice, microglia were further stained with antibodies against CD86 (T cell co-stimulator molecule B7.2), iNOS and CD16/32 (IgG receptors III and II, FcγR III/II), markers of M1 microglia/macrophages. While no positive signals of CD86 and iNOS were detected on microglia of any groups of mice (data not shown), the increase of CD16/32 expression on CD11b^+^ microglia was observed in dorsal horns of lumbar spinal cords in lean and DIO17 month-mice whereas only few CD16/32 positive cells were found in lean 3 month- and lean 6 month-mice (Fig. 3a). Those CD11b and CD16/32 double positive cells in 17 months aged mice were mostly localized in superficial laminae (laminae I and II) of dorsal horns where central afferents of sciatic nerves terminate. The expression of CD16/32 was further enhanced in obese aged (DIO 17 months) mice, in parallel to an increase of CD11b^+^ microglia numbers.

Microglia are also CNS professional phagocytes. We used an antibody against CD68 to determine the amount of glycoproteins expressed on the lysosomes of tissue macrophages. As a member of the lysosomal associated membrane glycoprotein (LAMP) family and also a member of the scavenger receptor family, the expression of CD68 can reflect, to some extent, the capability of microglia in removing intracellular (autophage) and extracellular (phagocytosis) debris. We found an increase of CD68 within spinal microglia in obese and aged mice, which anatomically, matched with intracellular aggregation of lipofuscin. Again obesity aggravated the effect of aging on CD68 expression. It appeared that the increase of CD68 expression occurred prior to the heap-up of lipofuscin within microglia (Fig. 3b).

### Aging and obesity associated inflammatory response within the spinal cords

**Fig. 4:**
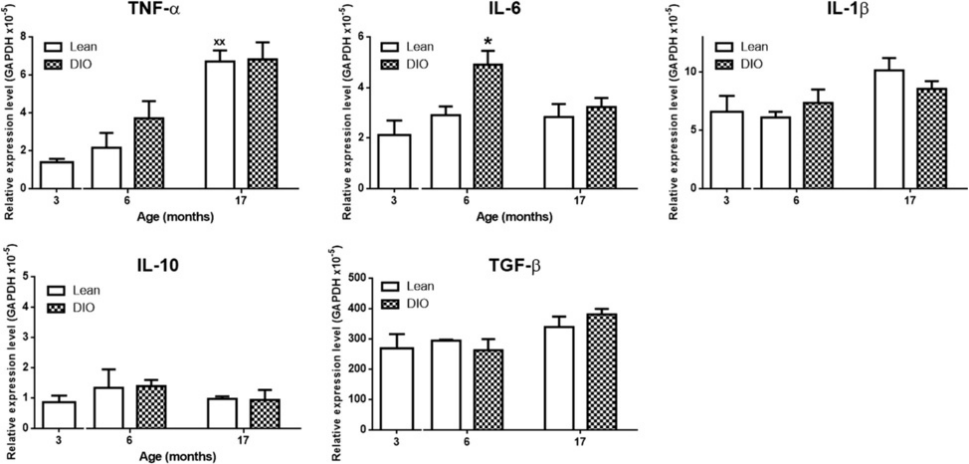
Inflammatory mediators in lumbar spinal cord. The mRNA levels of pro-inflammatory cytokine TNF-α was highly increased in lean 17 month-mice (*n* = 3/group, ^xx^ *p* < 0.01, lean 3 vs. lean 17). IL-6 mRNA expression was increased in DIO 6 mice (*n* = 3/group, *p < 0.05, lean 6 vs. DIO 6). IL-1β was not affected by aging nor by obesity (*n* = 3/group, *p* > 0.05). The expression of anti-inflammatory cytokines including IL-10 and TGF-β were not significantly altered

As immune competent cells in the CNS, microglia play major roles in generating inflammatory response in the spinal cords. Although some M1 pro-inflammatory markers were not detectable with immunohistochemistry method, the increase of pro-inflammatory cytokines in obese and aged spinal cords was found using real time PCR approaches. The mRNA levels of pro-inflammatory cytokine TNF-α was highly increased in lean 17 month-mice compared to lean 3 month-mice (*p* < 0.01). IL-6 mRNA expression was increased in DIO 6 month-spinal cords (*p* < 0.05). IL-1β was not affected significantly by aging nor by obesity (*p* > 0.05). Compared with lean 3 month-mice, the expression of anti-inflammatory cytokines including IL-10 and TGF-β were not significantly changed in all settings (lean 6 month-, DIO 6 month-, lean 17 month- and DIO 17 month-mice) (Fig. 4).

## Discussion

Abnormal pain behavior was observed in adult obese, aged lean and aged obese mice in the current study. More specifically, to compare with adult lean animals, obese and aged mice showed enhanced painful responses to heat and cold stimuli, however they became hyposensitive to mechanical von Frey stimulation. The impact of aging and obesity on microglia properties was evidenced by increased microglial cell density in the spinal cords, stereotypical morphological changes and polarization towards pro-inflammatory phenotype. Together with systemic inflammation, central inflammatory reaction mediated essentially by spinal microglia could contribute to altered pain responses in aged and obese mice.

As we observed in rodents, abnormal pain behavior has been reported in aging and obese human population. The prevalence of pain that impedes daily activities increases with age in the Spanish [21], Unites States [22] and Norwegian [23] population. Comparable prevalence was also found in Taiwanese communities [24] and in the Mediterranean region [6]. Several cross-sectional analysis demonstrated that obesity is strongly associated with chronic pain in general population [7, 25, 26], as well as in knee, hip and back pain of older people [27]. The Einstein Aging Study (EAS), a community-based study in the Bronx, demonstrated that body mass index (BMI) has a significant dose-dependent relationship with chronic pain [22]. The data from a longitudinal study [7] showed that odds of developing pain are higher in people with higher BMI or waist circumference at baseline. The link between obesity and pain has been attributed to greater load on weight-bearing joints, as many obese people reported lower limb and low back pain [28, 29]. However, obesity associated pain was not exclusively linked to weight-bearing structures, chronic pain in non-weight-bearing areas, including chronic head, neck, shoulder and abdominal pain have also been reported [25] . Metabolic syndrome [30] and limited mobility [31] have been considered as potential contributors to abnormal sensory behavior in obese people. One of the most plausible explanations for increased prevalence of chronic pain in obese and aging populations, especially in obese elderly, could be chronic inflammation. Indeed, both obesity and aging are associated with pro-inflammatory states where high levels of C-reactive protein, IL-6 and TNF were found in the blood [32, 33]. Whether and how central inflammation mediated by spinal microglia could contribute to altered sensory response in these populations remains to be elucidated.

Microglia turn-over is usually slow. They are long-lived cells in the CNS, which suggests that microglia could be susceptible to *in situ* aging effects occurring over a normal lifespan. In adult physiological conditions, microglial cells serve beneficial roles in the CNS. They act not only as immunological sentinels to fend off potentially dangerous insults, but also as constitutively neuroprotective glia that help sustain neuronal function in the CNS. In response to various local and/or systemic disturbances, they are stage-managers of CNS inflammatory responses. Microglia are also involved in synapse maintenance and elimination [34], clearance of apoptotic cells [35] and phagocytosis of cellular debris [36]. As aging is one of the largest risk factors for many diseases, aging associated changes in microglia have been postulated to drive pathological progression through a loss of, or reduced neuroprotection, an increase of neurotoxicity, and dysregulated response to internal/external signals [37, 38]. With normal aging, microglia in the brain exhibit de-ramified morphology, have increased mRNA and protein expression of several inflammatory markers, such as MHCII, CD68, CD11b/c, and release more pro-inflammatory cytokines [15, 39]. As microglia show remarkable anatomical, morphological and functional diversity in each area of the CNS [40], different functional organizations (e.g., brain, spinal cord and retina) harboring microglia have distinct activation thresholds that are primed to respond differently to insults. Although age-related changes in microglia have been explored in the brain and in the retina, very few, if any [41], of such studies with regard to the spinal cords have been found in the literatures. It is however of great value to determine spinal microglia phenotypes in aging and obese animals and patients, as the spinal cord is critical for the transmission of pain signals from periphery to the brain. Understanding aging and obesity related alterations of spinal microglia biology may illuminate potential contributions of this cell population to aging/obesity associated sensory disorders.

Our results revealed that aging has a profound effect on spinal microglia, which was exemplified by a significant increase in the number of microglia cells, hypertrophic cell bodies filling with lipofuscin and retracting/shortening processes. They displayed pro-inflammatory profile which was evidenced by enhanced expression of cytokine TNF-α and Fcγ receptors (CD16/32). All these aging-related changes on microglia have a consequence on disturbing spinal cord homeostasis and functional outcome. First, it has been well documented that pro-inflammatory cytokines in the spinal cords contribute to the central sensitization and pain hypersensitivity [42]. Second, cytokines or activated complement proteins released during pro-inflammatory reaction enhanced FcγR expression [43, 44], as we observed in microglia of lean/obese aged mouse spinal cords. Dysfunction of blood neural barriers reported in normal aged animals and in human [45, 46] could allow the entry of plasma proteins including IgG into the CNS parenchyma. The binding of Fcγ receptors on microglia to IgG antibodies not only links the powerful effector of the innate immune system, e.g. microglia, to the humoral immune response, but also links it to the adaptive immune responses via the antibody-antigen immune complexes. Such inflammatory reaction and antibody-dependent cell-mediated cytotoxicity can further increase spinal neurons excitability [47]. Third, aged microglia had shortened and less branched processes, which probably compromise their ability to survey and interact continuously with their environment, including modulating synaptic activities within the spinal cords. It has been shown that the response of senescent microglia to extracellular ATP and injury was dysregulated, usually slower in initiation, but sustained for longer periods of time [48]. Fourth, microglia in aging spinal cords were filled with a large amount of lipofuscins. Heavy accumulation of such undegradable cellular wastes can result in profound cellular dysfunction and damage, such as sensitizing cells to oxidative stress, interfering in phagocytic activity [20], which could lead to an alteration in local physiological processes. It appears that the increase of CD68 in aged mouse spinal microglia occurred prior to the accumulation of lipofuscins, and intracellular location of CD68 matched perfectly with the lipofuscin deposition. Such coincidence might suggest an increased autophage in aged mouse microglia, which could be responsible for lipofuscin accumulation. In addition to the fact that enhanced autophage and/or decreased lysosomal proteolytic activity can lead to intracellular lipofuscin aggregation, it has also been suggested that high concentration of lipofuscins in microglia might result from the phagocytosis of neurons with lipofuscin-loaded lysosomes [49]. The impact of obesity and/or metabolic syndrome on microglia was largely underexplored. It has been reported that high-fat-diet exposure induced IgG accumulation in hypothalamic microglia [50]. Microglia morphology was found altered in obese mice deficient of Ifitm (Interferon-induced transmembrane gene family) proteins [17]. Obesity in aging exacerbates blood brain barrier dysfunction, neuroinflammation and oxidative stress in mouse hippocampus [51]. In line with the findings from the aged brain, our study on the spinal cords of aged and obese mice revealed that obesity remarkably aggravated the damage caused by aging on microglia, including a significant increase of microglia cell density, profound changes on microglia morphology and lipofuscin accumulation. Such damage can synergistically connect obese elderly to sensory impairment.

On the whole, microglia cell reactivity has important consequences in preserving adult neuronal structure and function [52]. Aging/obesity associated microglia changes in the spinal cord dorsal horns can dysregulate microglia-neuron crosstalk and impair physiological pain signal transmission. The inflammatory functions of microglia have special relevance for understanding of abnormal pain behavior in aged populations. It is, therefore, of importance to identify strategies (e.g., dietary or exercise intervention) to restore microglial cells in the elderly and/or obese brain/spinal cord to its healthy youthful state.

## Materials and methods

### Animals

C57BL/6 mice (males, 10-12 weeks old, 20–25 g) were purchased from the Charles River Laboratories. All animals were acclimatized to standard laboratory conditions (14-h light, 10-h dark cycle) and given free access to rodent chow and water. For lean mice, C57BL/6 mice were kept up to 6 or 17 months while feeding them regular diet containing 10 % kcal fat, 70 % kcal carbohydrate and 20 % kcal protein (Research Diets, Inc. D12450B). Diet-induced obese (DIO) mice were induced by feeding C57BL/6 mice with high-fat diet containing 60 % kcal fat, 20 % kcal carbohydrate, 20 % kcal protein (Research Diets, Inc. D12492). Special diet started from the age of 3 months and kept until the age of 6 or 17 months. Animal body weight was monitored once per month and blood glucose levels were measured using a hand-held glucometer (Contour, Bayer) following over night fasting. C57BL/6 mice aged 10-12 weeks were used as theoretical baseline control. All mice were housed in 2–4 mice/cage. All experiments were approved by the Institutional Animal Care and Use Committee of McGill University (Permit #5990) and conformed to the ethical guidelines of the International Association for the Study of Pain.

### Behavioral analysis

Mice were habituated to the testing environment 1 to 2 h daily for at least two days before baseline testing.

Rotarod assay was used to assess mouse motor coordination. The task included a speed ramp from 0 to 30 rpm over 60 s, followed by an additional 240 s at the maximal speed. The time that each animal walked on the rod before falling was recorded.

Mechanical sensitivity was assessed with calibrated von Frey hairs (Stoelting) using the up-down method [53]. Mice were placed on a metal mesh floor with small Plexiglas cubicle containers and allowed at least 1 h for habituation before testing. A set of eight calibrated von Frey filaments with increasing stiffness (ranging from 0.008 to 1.40 g of force) were applied to the plantar surface of the hindpaw until they bent. A positive reaction was recorded if mice exhibited a brisk paw withdrawal reaction from the stimuli. The threshold force required to elicit withdrawal of the paw (median 50 % paw withdrawal) was determined as the average of two tests separated by at least 1 h.

Acetone test was performed on mice hind paws to evaluate cold allodynia. Mice were placed in the same setting described above for the von Frey test. Total duration of acetone evoked behaviors (flinching, licking or biting of their hind paws) was counted during 1 min after one drop of acetone (~25 μl) application to the plantar surface of the hindpaw.

Hot plate (55 °C) was used as an unpleasant sensory heat stimulus to measure thermal hypersensitivity. The latency to paw-licking, squeaking, or distressful behaviors was measured.

### Tissue preparation

Mice were deeply anesthetized with a ketamine/xylazine mixture. Anesthetized mice were perfused transcardially with 0.9 % NaCl. Lumbar parts (L4 to L6) of spinal cords were extracted and immediately stored in -80 °C until use (for real time (RT)-quantitative PCR analysis). For histological studies, mice after 0.9 % NaCl transcardial perfusion were further perfused with 4 % paraformaldehyde (PFA) in 0.1 M sodium phosphate buffer at pH 7.4. Lumbar spinal cords were removed and placed in 4 % PFA overnight and then transferred to 30 % sucrose for cryoprotection in -20 °C. Frozen lumbar spinal cords were cut transversely into 25 μm-thick sections on a sliding microtome and collected in an anti-freeze solution (0.05 M sodium phosphate buffer containing 30 % ethylene glycol and 20 % glycerol, pH7.3).

### Immunohistochemistry

A standard fluorescent immunohistochemistry (IHC) protocol was applied to characterize spinal microglia phenotypes. Free-floating sections were incubated in a blocking buffer consisting of 3 % goat or rabbit serum, 1 % bovine serum albumin, and 0.25 % Triton X-100 in Tris-buffered saline (TBS) for 1 h at room temperature followed by overnight primary antibody incubation at 4 °C. Sections were then incubated with Alexa Fluor 488, 594 or 647-conjugated secondary antibodies (1:500; Invitrogen) for 1 h at room temperature followed by 5 min incubation of 4′, 6-diamidino-2-phenylindole (DAPI) for nuclear counterstaining (1:100,000; Sigma). Sections were mounted onto slides, then coverslipped with Vectashield mounting medium (Vector Laboratories). Rabbit anti-ionized calcium-binding adaptor molecule-1 (Iba-1) polyclonal primary antibody (1:1000; Wako Chemicals) followed by Alexa Fluor 594-conjugated goat anti-rabbit secondary antibody was used for microglia quantitative and morphological analysis. Double immunostaining was performed using rat anti-CD11b (1:500; AbD Serotec) and goat anti-CD16/32 (1:200; R&D systems) primary antibodies followed by Alexa Fluor 488-conjugated rabbit anti-rat and 647-conjugated rabbit anti-goat secondary antibodies respectively. Rabbit Iba-1 and Rat anti-CD68 (1:500; AbD Seroect) primary antibodies were labeled with Alexa Fluor 594-conjugated goat anti-rabbit and Alexa Fluor 647-conjugated goat anti-rat secondary antibodies respectively.

### Image processing and analysis

The immunofluorescent staining was examined under microscope. Images were acquired using an Olympus BX51 microscope equipped with a color digital camera (Olympus DP71). Quantitative analysis of the immunofluorescent Iba-1^+^ cells was performed on images digitized using a constant set of parameters (exposure time, gain and post-image processing) with special attention to avoid signal saturation. Images were acquired using 20x objectives of the microscope, then the area of interest (AOI) was placed around superficial laminae (laminae I and II) in dorsal horn (DH) of lumbar spinal cords, then, Iba-1^+^ cells were assessed by manual tracking of microglia-shaped signals in red co-stained with blue DAPI nuclei. Six to ten sections per animal were used.

### RNA extraction and RT-PCR

Tissue homogenization was performed with tissue homogenizer beads and a Precellys 24 tissue homogenizer (Bertin Technologies) using two 20s pulses at 5600 rpm. Total RNA was extracted from lumbar spinal segments using single-step RNA isolation method with TRIzol® Reagent (ambion by Life technologies) following manufacturer’s protocols. The ratio of absorbance at 260 and 280 nm was measured by a nanodrop 2000 (Thermo Scientific) to assess purity and concentration of RNA. cDNA synthesis was performed using 1 μg of total RNA with SuperScript III Reverse Transcriptase (Invitrogen, Burlington, ON). qPCR primers were synthesized by Integrated DNA Technologies, and their specificity was verified using the GenBank database from the National Center for Biotechnology Information website. Detailed information on primers is shown in Table 1. SYBR Green mix was obtained from Bio-Rad (iTaq, Hercules, CA). qPCR was performed using a Rotor-Gene Q real-time PCR cycler (Qiagen, Toronto, ON). Data were interpreted using the comparative Ct method [54]. All cytokine data were compared to endogenous glyceraldehyde 3-phosphate dehydrogenase.

**Table 1:** Detailed information on the selection of primers for real-time reverse-transcriptase polymerase chain reaction

Gene	NCBI ID #	Forward		Reverse	
		Primer	Sequence	Primer	Sequence
IL1β	NM_008361.3	mIL1β-F1	CTATACCTGTCCTGTCTA	mIL1β-R1	GCTCTTGACTTCTATCTTG
IL6	NM_031168.1	mIL6-F1	CTGAAACTTCCAGAGATA	mIL6-R1	TTCATGTACTCCAGGTAG
TNFα	NM_013693.2	mTNFα-F1	TTCTGTCTACTGAACTTC	mTNFα-R1	CCATAGAACTGATGAGAG
IL10	NM_010548.2	mIL10-F1	CTATGCTGCCTGCTCTTA	mIL10-R1	GCTGGTCCTTTGTTTGAAA
TGFβ	NM_009367.3	mTGFβ-F1	AGAGAAGAACTGCTGTGT	mTGFβ-R1	GGTTGTGTTGGTTGTAGAG
GAPDH	NM_008084.2	mGAPDH-F1	GTGAAGGTCGGTGTGAAC	mGAPDH-R1	AATCTCCACTTTGCCACTG

*NCBI* National Center for Biotechnology Information

*IL* interleukin

*TNF* tumor necrosis factor

*TGF* transforming growth factor

*GAPDH* glyceraldehyde 3-phosphate dehydrogenase

### Statistical analysis

All data were presented as mean ± SEM. For all statistical analysis, unpaired t-tests were performed to measure differences in pain behaviors, microglia numbers and inflammatory mediator levels between i) lean 6 month- and DIO 6 month-mice, ii) lean 17 month- and DIO 17 month-mice, and iii) DIO 6 months and DIO 17 months aged mice. One-way ANOVA followed by Dunnett’s test was performed to compare differences among lean animals (3, 6, 17 months aged mice). The criterion for statistical significance was *p* < 0.05.
